# Fuzi polysaccharides improve immunity in immunosuppressed mouse models by regulating gut microbiota composition

**DOI:** 10.1016/j.heliyon.2023.e18244

**Published:** 2023-07-13

**Authors:** Ran Tu, Cheng Zhou, Wenfeng Huang, Zhengping Feng, Qiufang Zhao, Xiaofei Shi, Langjun Cui, Keke Chen

**Affiliations:** a Medical Laboratory of Jingmen People's Hospital, Jingchu University of Technology Affiliated Central Hospital, Jingmen, Hubei, China; b National Engineering Laboratory for Resource Development of Endangered Crude Drugs in Northwest China, Key Laboratory of Medicinal Resources and Natural Pharmaceutical Chemistry, Ministry of Education, College of Life Sciences, Shaanxi Normal University, Xi'an, Shaanxi, China; c Yan'an Hospital of Traditional Chinese Medicine, Yan'an, Shaanxi, China; d School of Biological and Environmental Engineering, Xi'an University, Xi'an Key Laboratory of Natural Product Development and Anticancer Innovative Drug Research in Qinling, Xi'an, Shaanxi, China

**Keywords:** 16S rRNA, Fuzi polysaccharides, Gut microbiota, Immunosuppressive mouse model, Short-chain fatty acids

## Abstract

**Rationale and objectives:**

Fuzi, the dried root of *Aconitum carmichaelii* Debx, is one of the widely used traditional Chinese medicines. Fuzi polysaccharides are considered the most bioactive compounds with immunomodulatory functions, however, the mechanisms have not been evaluated. This study aims to systematically investigate the effects of Fuzi polysaccharides on the gut microbiota and immune function using a mouse model immunosuppressed with cyclophosphamide.

**Methods:**

The short-chain fatty acid levels in cecal contents were measured by gas chromatography-mass spectrometry. The gut microbiota 16S rRNA gene were sequenced by next generation sequencing. The mRNA expression levels of NF-κB, IL-6, TNF-α, iNOS and COX-2 were measured using quantitative real-time polymerase chain reaction. The protein expression of occludin and zonula occludens-1 were analyzed by Western blot. The white blood cells were counted using automated hematology analyzer, and CD4^+^FOXP3^+^/CD4^+^ ratio was measured by flow cytometry.

**Results and Conclusions:**

Fuzi polysaccharides had the function of elevating the concentration of acetic acid, propionic acid, isobutyric acid, and n-butyric acid in the cecum. Meanwhile, Fuzi polysaccharides could decrease the relative abundance of *Helicobacter*, *Anaerotruncus*, *Faecalibacterium*, *Lachnospira*, *Erysipelotrichaceae*_UCG-003, *Mucispirillum*, and *Mycoplasma*, and increase the relative abundance of *Rhodospirillales*, *Ruminococcaceae*_UCG-013, *Mollicutes*_RF39, *Ruminococcus*_1, *Christensenellaceae*_R-7_group, and *Muribaculaceae* in the gut. Furthermore, Fuzi polysaccharides exhibited the function of increasing spleen and thymus indices and number of white blood cells and lymphocytes. Fuzi polysaccharides could reverse the decreased mRNA expression of NF-кB, IL-6, and iNOS, differentiation of CD4^+^FOXP3^+^ regulatory T cells as well as protein expression of occludin and zonula occludens-1 induced by cyclophosphamide. In addition, the mRNA and protein expression of cytokines were significantly correlated with the abundance of gut microbiota under Fuzi polysaccharides treatment. Collectively, the above results demonstrated that Fuzi polysaccharides could regulate inflammatory cytokines and gut microbiota composition of immunosuppressive mice to improve immunity, thereby shedding light on revealing the molecular mechanism of polysaccharides of traditional Chinese medicines in the future.

## Highlights

• Fuzi polysaccharides increase levels of short-chain fatty acids.
• Fuzi polysaccharides regulate intestinal microbiota composition/distribution.
• Fuzi polysaccharides increase number of white blood cells and lymphocytes.
• Fuzi polysaccharides increase inflammatory cytokines and T cell differentiation.
• Fuzi polysaccharides can improve immunity in immunosuppressed mouse models.

## Graphical abstract

**Figure:**
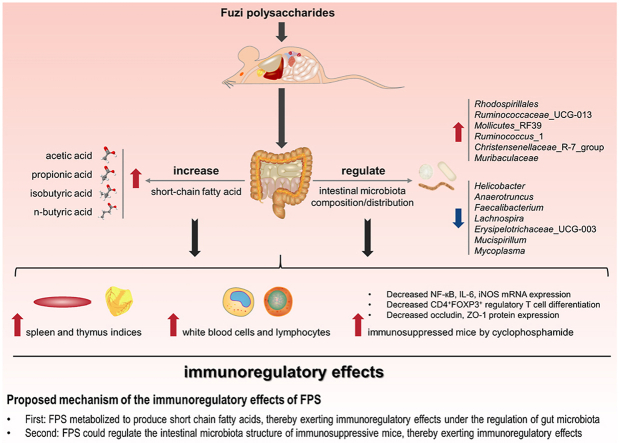

## 1 Introduction

In recent years, immunosuppression has become a serious clinical issue that increases the incidence and severity of many infectious diseases (e.g., the administration of immunosuppressant drugs given for various medical conditions). The underlying diseases includes Human Immunodeficiency Virus (HIV) infection or malignancy (e.g., lymphoma) which affects the immune system producing secondary immunodeficiency, and various primary immunodeficiency syndromes such as severe combined immunodeficiency (SCID) with defects in T cells, B cells, and NK cells [1], especially under the current and post SARS-CoV-2 pandemic [2]. For instance, recent cohort study using data from the National COVID Cohort Collaborative found that 16,494 patients (7%) had active medication records for immunosuppressive medications at the time of admission, including medications commonly used for a rheumatological condition (5366 [33%] patients), antimetabolite drugs (4288 [26%]), or for cancer treatment (3569 [22%]) among hospitalized adults. Meanwhile, the traditional Chinese medicines has been found to play key immunoregulatory roles in a series of immune-related diseases [3]. Considering this, it is promising to solve the above issues from the perspective of bioactive ingredients in traditional Chinese medicines.

The processed lateral root of *Aconitum carmichaelii* Debeaux, known as “Fuzi” in China, is a famous traditional Chinese medicine widely used to treat rheumatism, cardiovascular diseases, joint pain, syncope, and bronchial asthma [4]. Traditionally, Fuzi extracts are prepared as medicinal slices, decoction and oral liquids for consumption in households. Hei-Shun-Pian, the most common type of Fuzi, is combined with other traditional Chinese medicines including *Radix ginseng*, *Radix glycyrrhizae*, and *Zingiberis rhizoma*. Previous studies have shown that Hei-Shun-Pian could improve blood circulation and is used to treat heart failure, neuralgia, rheumatism, gout, hepatic diseases, and endocrine disorders [[5], [6], [7]]. Moreover, Fuzi is used to prevent colds in several regions (e.g., Shaanxi Province, Sichuan Province, Yunnan Province, etc.) in China [8]. In recent decades, several major diseases including cardiovascular diseases, chronic kidney diseases and neurodegenerative diseases have been significant health problems globally [9,10]. Recently, a series of studies showed that Fuzi products including Hei-Shun-Pian, Bai-Fu-Pian and other traditional Chinese medicines had anti-inflammatory and immune-enhancing effects in such major diseases. For instance, Fuzi-Lizhong decoction, composed of Fuzi, *Codonopsis pilosula* (Franch.) Nannf., *Atractylodes macrocephala* Koidz., *Zingiber officinale* Roscoe, and *Glycyrrhiza uralensis* Fisch., improved immunity in rats by decreasing the levels of IL-2, IL-6, IL-10, and TNF-α [11]. Fujiang oral liquid, a combination of Fuzi and *Z*. *officinale*, increased the immune organ index, the activity of natural killer (NK) cells, and the number of antibody-forming cells in the spleen of normal and immunosuppressed mice [12]. In addition, it is demonstrated that single dose extract of *A*. *carmichaelii* could modulating adaptive immunity and natural killer-related immunity in mice [13].

The improvement in immune function by Fuzi is attributed to polysaccharides and aconitine, while the anti-inflammatory and immunosuppressive activity originates from N-methyl-pseudoephedrine, pseudoephedrine, benzoyl hypaconine, benzoylaconine, and mesaconine [14]. Specifically, Fuzi polysaccharide (FPS) is found to stimulate murine lymphocyte proliferation induced by concanavalin A and lipopolysaccharide *in vitro* and *in vivo* [15]. FPS can increase spleen and thymus indices, white blood cell count, NK cell activity, phagocytic activity of peritoneal macrophages, and antibody-forming ability and transformation of lymphocytes in immunosuppressed mice [16]. Moreover, FPS has the function of inducing the differentiation of peripheral blood mononuclear cells to dendritic cells and the proliferation of dendritic cells [17]. These findings have shown the immunomodulatory effects of Fuzi, however, the functional pathways and mechanisms of FPS therein still remains unclear.

Recent studies have identified gut microbiota as a key target of bioactive ingredients in traditional Chinese medicines. The gut microbiota is the collection of microorganisms in the digestive system of animals and humans. This microbial ecosystem produces several metabolites from the anaerobic fermentation of undigested dietary components and endogenous compounds in the colon [18]. The molecular basis of host-microbiome interactions is mediated by cell-to-cell communication and microbial metabolites [19]. Moreover, gut microbes influence host bile acid pools, which in turn modulate the microbial community structure. Microbial metabolites mediate the cross-talk between immune cells and the intestinal epithelium [20]. These metabolites participate in the metabolic reprogramming of immune cells and the epigenetic regulation of gene expression in these cells [19]. Several metabolites including vitamins, minerals, fatty acids, peptides, amino acids, and polyphenols affect immune homeostasis [21]. Specifically, polysaccharides present in traditional Chinese medicine concoctions affect the gut microbiota and host immunity. For instance, longan (*Dimocarpus longan* Lour.) polysaccharides changed the intestinal microbiota and gut metabolites, improving host immune function under stress conditions [22]. Water-soluble polysaccharides and concentrated alkali-soluble polysaccharides from purple sweet potatoes increased the spleen index, decreased the serum levels of IL-2 and IL-6, and increased the abundance of *Bacteroidetes*, *Lachnospiraceae*, and *Oscillospira* in cyclophosphamide treated mice [23]. *Hericium erinaceus* polysaccharide promoted the growth of beneficial gut bacteria and improved immunity in a rat model of inflammatory bowel disease [24]. These results thus raised the possibility that FPS in Fuzi could regulate immune functions via gut microbiota.

**Fig. 1:**
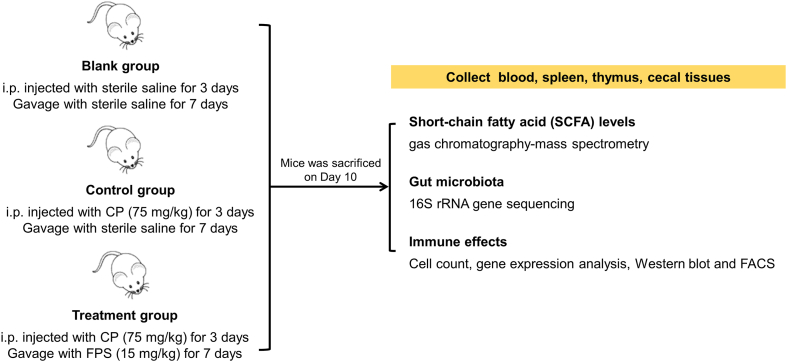
Flowchart of research methodology used in this study.

Based on the above assumptions, in this study we intend to first reveal the pharmacological effects of FPS and the mechanisms underlying these effects using *in vivo* immunosuppressed mice models with focus on the same bacteria existed in human (Fig. 1). Collectively, the aims of this study are: (1) to demonstrate the effects of FPS on gut microbiota composition; (2) to elucidate the effects of FPS on immunity; (3) to reveal the relationship between gut microbiota and immunity under FPS treatment.

## 2 Materials and methods

### 2.1 Preparation of FPS

Hei-Shun-Pian (the lateral root of *Aconitum carmichaelii* Debeaux processed using traditional methods) was purchased from Shaanxi Huake Biotech Company, Xi'an, China. The key indicator ingredient aconitine was quantified by high-performance liquid chromatography (Fig. S1 a, b, c). FPS was extracted as described previously [25]. Hei-Shun-Pian were milled (FW177 pulverizer; Taiste, Tianjin, China) and passed through 100-mesh sieves to obtain Hei-Shun-Pian powder. Hei-Shun-Pian powder (100 g) was extracted with 4000 mL of distilled water at 90 °C for 2 h with regular stirring. The extract was centrifuged at 3500 *g* for 15 min at 4 °C (Sorvall RC 3C Plus centrifuge; Thermo Scientific, USA), and the supernatant and the sediment were collected. The sediment was re-extracted twice. The supernatants were collected and concentrated under vacuum at 65 °C (RE52AA rotary evaporator; Yarong, Shanghai, China) and precipitated with 95% ethanol (1:3, v/v) at 4 °C for 24 h. The precipitate was dried under vacuum (LGL-6050 vacuum freeze drier; Axiuluo, Chongqing, China) to obtain crude FPS. Crude FPS was loaded onto a DEAE Sepharose Fast Flow column (2.6 cm × 30 cm; Chalfont, St. Giles, UK) and eluted with 0.125–0.5 M NaOH (Sinopharm Chemical Reagent Co., Ltd., Shanghai, China). The first eluted fraction containing pure FPS was collected (DBS-160-LCD Automatic Collecter; Qite, Shanghai, China), concentrated, dialyzed, and lyophilized (Fig. S1 d, e).

### 2.2 Animals and experimental design

The animal experiments conformed to the guidelines of the Animal Care and Use Committee of our institution. Kunming mice were purchased from the Laboratory Animal Center of Xi'an Jiaotong University Health Science Center (License key SCXK [Shan] 2014-001) and maintained under a 12 h light 12 h dark cycle at 22 ± 2 °C, with free access to food and water. After one week of adaptation, eight-week-old female mice were randomly divided into three groups of five animals under treatment conditions determined in preliminary studies as following: blank group, treated with sterile saline (Huaren Pharmaceutical [Rizhao] Co., Ltd, Shandong); control group, intraperitoneally injected with cyclophosphamide (Aladdin Biochemical Technology Co., Ltd., Shanghai, China) (75 mg kg^−1^ per day) for 3 days and gavaged with sterile saline for 7 days (Fig. S2); treatment group, intraperitoneally injected with cyclophosphamide (75 mg kg^−1^ per day) for 3 days and gavaged with FPS (15 mg kg^−1^ per day) for 7 days (Fig. S3). On day 10, the animals were anesthetized with an intraperitoneal injection of chloral hydrate (0.1 mL) (Sinopharm Chemical Reagent Co., Ltd., Shanghai, China) and weighed. Peripheral blood was collected from the heart and transferred to tubes containing dipotassium EDTA (Jiangsu Kangjie Medical Device Co., Ltd., Jiangsu, China), and the spleen and thymus were removed and weighed (Sartorius, Germany). Finally, the spleen index and thymus index were calculated and evaluated as follows, respectively:

Spleen index = spleen weight (mg)/body weight (g)

Thymus index = thymus weight (mg)/body weight (g)

Part of fresh spleen tissues was used to flow cytometry assay, the remainder spleen tissues, thymus tissues, cecal tissues and contents were collected in sterile centrifuge tubes (Eppendorf, Germany) and stored at −80 °C until further analysis. All animal experiments were approved by the animal research ethics committee of Xi'an University (No. 2020-4).

### 2.3 Measurement of short-chain fatty acid (SCFA) levels

SCFA levels in cecal contents were measured by gas chromatography-mass spectrometry, as described previously [26]. Cecum contents (0.1 g) were mixed with 0.5 mL of deionized water and shaken for 3 min to form a suspension. The pH was adjusted to 2–3 using HCl (Sinopharm Chemical Reagent Co., Ltd., Shanghai, China), and the suspension was centrifuged at 12,000 *g* for 20 min (Hermle, Germany). Then, 2-ethylbutyric acid (Sigma-Aldrich, St Louis, MO, USA) solution was added to the supernatant to a final concentration of 1 mM and served as an internal standard. Acetic acid, propionic acid, isobutyric acid, and n-butyric acid (Sigma-Aldrich, St Louis, MO, USA) served as external standards.

Chromatographic analysis was performed using a Thermo Trace 1300 GC system equipped with a flame ionization detector and a fused-silica capillary column (30 m × 0.25 mm internal diameter; J&W Scientific, Agilent Technologies Inc., USA) coated with a free fatty acid phase. The injected sample volume was 1 μL, and helium was the carrier gas. MS data were acquired using Xcalibur software. Each experiment was repeated independently at least three times.

### 2.4 Gut microbiota 16S rRNA gene sequencing

Total microbial genomic DNA from cecal contents was extracted using the QIAamp DNA Stool Mini Kit (Cat. No. 51504; Qiagen, Hilden, Germany). The V3–V4 regions of the bacterial 16S rRNA gene were amplified using forward primer 341F (5′-CCTACGGGNGGCWGCAG-3′) and reverse primer 805R (5′-GACTACHVGGTATCTAATCC-3′) by Annoroad Gene Technology Co., Ltd (Beijing, China) [27,28]. PCR was carried out 15 μL of High-Fidelity PCR Master Mix (Cat. No. R040A; TaKaRa, Japan), 1.5 μL of each primer, 10 ng of DNA template, and 12 μL of double-distilled water. PCR products were purified using the QIAquick PCR Purification Kit (Cat. No. 28104; Qiagen, Hilden, Germany). The barcoded V3–V4 PCR amplicons were sequenced on an Illumina MiSeq platform (Illumina, SD, USA). Genomic DNA extraction and gene amplification and sequencing were performed by Annoroad Gene Technology Co., Ltd (Beijing, China). Five samples from each group were sequenced.

Bioinformatics analysis was performed using the Biomarker Biocloud platform (http://www.biocloud.org). High-quality sequences with ≥97% similarity were clustered into operational taxonomic units (OTUs) using Usearch. OTUs were assigned to bacterial taxa using the SILVA database (Release132, http://www.arb-silva.de) [29]. Principal coordinate analysis (PCoA) and unweighted pair-group method with arithmetic mean analysis were performed using R software.

### 2.5 RNA extraction and mRNA gene expression analysis

Total RNA of spleen tissue was isolated using TRIzol (Invitrogen, USA), as described previously [30], and reverse-transcribed into cDNA using the PrimeScript RT Reagent Kit (Cat. No. RR047A; TaKaRa, Japan). Quantitative real-time polymerase chain reaction (qRT-PCR) was performed using 1 μL of cDNA, 1 μL of each primer, 10 μL of SYBR Premix (Cat. No. RR420A; TaKaRa, Japan), and 7 μL of double-distilled water. Amplification conditions consisted of an initial denaturation at 94 °C for 5 s followed by 40 cycles at 60 °C for 35 s and 72 °C for 60 s. The mRNA gene expression was normalized to the housekeeping gene GAPDH using the 2^-ΔΔCT^ method [31]. The primers used are shown in Table S1.

### 2.6 Western blot analysis

Western blot analysis was conducted according to protocols in previous studies [32,33]. Cecal tissues (100 mg) were homogenized (QIAGEN, Germany) in protein extraction buffer. Lysates were fractionated on a 10% sodium dodecyl sulfate-polyacrylamide gel (Cat. No. P1200; Solarbio, China) and electroblotted onto polyvinylidene difluoride membranes (Millipore, USA). Membranes were blocked with 5% non-fat milk (Cat. No. D8340; Solarbio, China) at room temperature for 1 h, probed with primary antibodies against occludin (1:250, ab222691; Abcam) and zonula occludens-1 (ZO-1) (1:250, ab216880 m; Abcam) at 4 °C overnight and then incubated with horseradish peroxidase-conjugated secondary antibody (anti-rabbit IgG, H + L; 1:2000–1:20,000; ab6721, Abcam). Immunoreactive bands were visualized using an enhanced chemiluminescence detection system (Millipore, USA).

### 2.7 Cell count

The number of white blood cells in the peripheral blood was counted using an automated hematology analyzer (KX-21, Sysmex Corporation, Japan). Fresh spleen tissues were rinsed in saline, minced into fine pieces and grinded with syringe core simultaneously wash with cooled PBS buffer (pH 7.4) passed through a 40 μm filter. Added cooled PBS buffer (pH 7.4) to 40 mL, inverted severs times and brief centrifugation (1000 g). Added 5 mL red blood cell lysis buffer to the pellets cells and lysed for 10 min at room temperature and resuspended in cooled PBS buffer (pH 7.4). Cells were stained with anti-mouse CD4 (Cat. No. 100401) and FOXP3 (Cat. No. 320001) antibodies (Biolegend, USA) or the matching control isotypes for 1 h at 4 °C in the dark. The cells were rinsed twice and resuspended in cooled PBS buffer (pH 7.4). Fluorescence was measured by flow cytometry using a Guava easyCyte HT system (Millipore, USA), and data were analyzed using FlowJo software. Each experiment was repeated independently three times.

### 2.8 Statistical analysis

Statistical analyses were performed by one-way analysis of variance, followed by Tukey test using R version 4.0.1 (http://www.r-project.org/). *P*-values of less than 0.05 were considered statistically significant.

## 3 Results

### 3.1 FPS treatment increased SCFA production in immunosuppressed mice

**Fig. 2:**
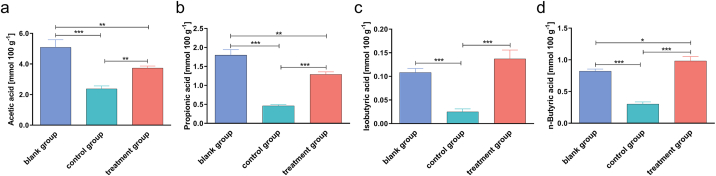
Relative concentration of short-chain fatty acids (SCFA) in the cecal contents of control and treated mice. (a) Acetic acid. (b) Propionic acid (c) isobutyric acid. (d) n-butyric acid. Data are expressed as mean ± SD (five animals per group, n = 5). *p < 0.05, **p < 0.01, ***p < 0.001 by one-way analysis of variance.

To elucidate the effects of FPS on SCFA production, the contents of acetic acid, propionic acid, isobutyric acid, and n-butyric acid in cecum were estimated and compared among blank group, control group and treatment group in mice. The results showed that there exists a coincident trend regarding the effects of FPS on the production of the four SCFAs including acetic acid (Fig. 2a), propionic acid (Fig. 2b), isobutyric acid (Fig. 2c) and n-butyric acid (Fig. 2d). In control group, the contents of the four products were lower than those in blank group (*p* < 0.001). While in treatment group, these contents were higher compared with control group (*p* < 0.01). Collectively, the above results indicated that FPS treatment increased SCFA production in immunosuppressed mice.

### 3.2 FPS treatment significantly altered the gut microbiota composition

The effects of FPS on gut microbiota composition were further assessed by analyzing the 16S rRNA gene (V3–V4 region) in cecal samples. After removing low-quality sequences, 1,317,284 clean reads were generated, and each sample (n = 5 for each group) produced an average of 87,819 ± 38,206 high-quality reads. Rarefaction and Shannon curves showed that the sequencing depth covered new phylotypes and most bacterial phyla (Fig. S4). The most abundant phyla were *Firmicutes* and *Bacteroidetes*; however, the abundance of *Firmicutes* and *Bacteroidetes* varied across groups (blank group: 59.68 ± 4.48 and 31.77 ± 5.51; control group: 41.60 ± 8.34 and 22.34 ± 6.14; treatment group: 55.85 ± 6.14 and 35.39 ± 5.32). Moreover, *Epsilonbacteraeota*, *Deferribacteres*, *Proteobacteria*, and *Actinobacteria* were highly represented in these samples (Fig. S5).

**Fig. 3:**
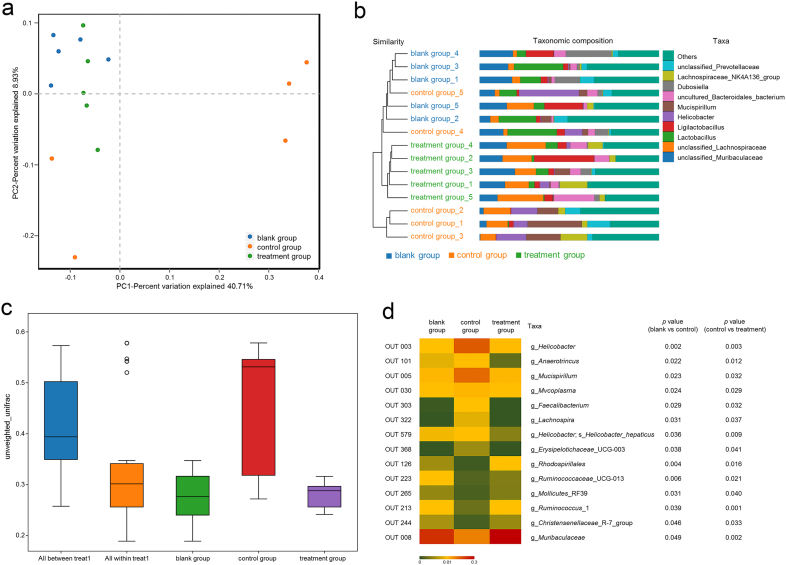
Gut microbiota composition in control and treated mice. (a) Principal component analysis scores based on the relative abundance of operational taxonomic units (OTUs) (97% similarity level). (b) Unweighted pair-group method with arithmetic mean analysis. (c) Analysis of similarities. (d) Bacterial genera in which the relative abundance of OTUs was significantly altered by Fuzi polysaccharides based on Metastats analysis. Nonparametric *t* tests (Mann-Whitney). Data are expressed as mean ± SD (five animals per group, n = 5). *P*-values of less than 0.05 were considered statistically significant.

UniFrac-based PCoA revealed that the three groups clustered independently (Fig. 3a). In contrast, the analysis of unweighted pair-group method with arithmetic mean and similarities revealed that the microbial composition of the treatment group was more similar to that of the blank group (Fig. 3b and c).

Bacterial phylotypes significantly altered by FPS treatment were identified by Metastats analysis. FPS decreased the abundance of seven genera (*Anaerotruncu*s, *Faecalibacterium*, *Lachnospira*, *Erysipelotrichaceae*_UCG-003, *Helicobacter*, *Mucispirillum*, and *Mycoplasma*) and increased the abundance of six genera (*Ruminococcaceae*_UCG-013, *Ruminococcus*_1, *Christensenellaceae*_R-7_group, *Rhodospirillales*, *Mollicutes*_RF39, and *Muribaculaceae* (Fig. 3d).

### 3.3 FPS treatment reversed decreasing spleen and thymus indices and number of white blood cells and lymphocytes by cyclophosphamide

**Fig. 4:**
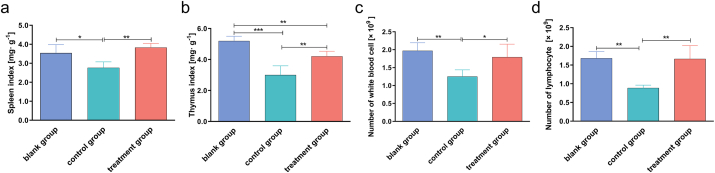
Spleen index, thymus index, number of white blood cells, and number of lymphocytes in control and treated mice. (a) Spleen index. (b) Thymus index. (c) Number of white blood cells. (d) Number of lymphocytes. Data are expressed as mean ± SD (five animals per group, n = 5). *p < 0.05, **p < 0.01, ***p < 0.001 by one-way analysis of variance.

To demonstrate the immunoregulatory effect of FPS, the spleen index, thymus index, number of white blood cells, and number of lymphocytes were estimated in control and treated mice. The results showed that cyclophosphamide significantly decreased the spleen and thymus indices (Fig. 4a and b) and the number of white blood cells and lymphocytes (Fig. 4c and d) (*p* < 0.05). On the contrary, these effects were reversed in FPS treat group, exhibiting an increasing trend of spleen and thymus indices and number of white blood cells and lymphocytes compared with cyclophosphamide group. These results indicated that FPS had the function of enhancing immunity in immunosuppressed mice and the molecular mechanism are needed to further investigated.

### 3.4 FPS treatment reversed decreasing cytokine expressions and CD4^+^FOXP3^+^/CD4^+^ ratio by cyclophosphamide

**Fig. 5:**
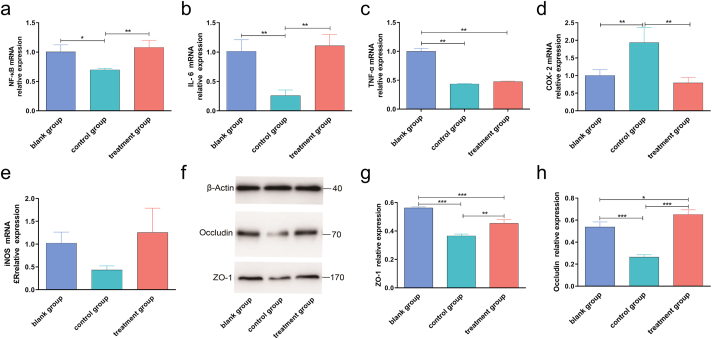
Relative mRNA expression of cytokines (NF-κB, IL-6, TNF-α, COX-2, and iNOS) and expression of tight junction proteins (occludin and ZO-1) in control and treated mice. (a) mRNA expression of NF-κB. (b) mRNA expression of IL-6. (c) mRNA expression of TNF-α. (d) mRNA expression of COX-2. (e) mRNA expression of iNOS. (f) Representative western blotting images of tight junction proteins (ZO-1 and occludin). (g) Protein expression level of ZO-1. (h) Protein expression level of occludin. The mRNA expression in spleen tissues was assessed by quantitative real-time polymerase chain reaction, and protein expression in cecal tissues was evaluated by western blotting. β-Actin was used as a loading control. Data are expressed as mean ± SD (five animals per group, n = 5). *p < 0.05, **p < 0.01, ***p < 0.001 by one-way analysis of variance.

**Fig. 6:**
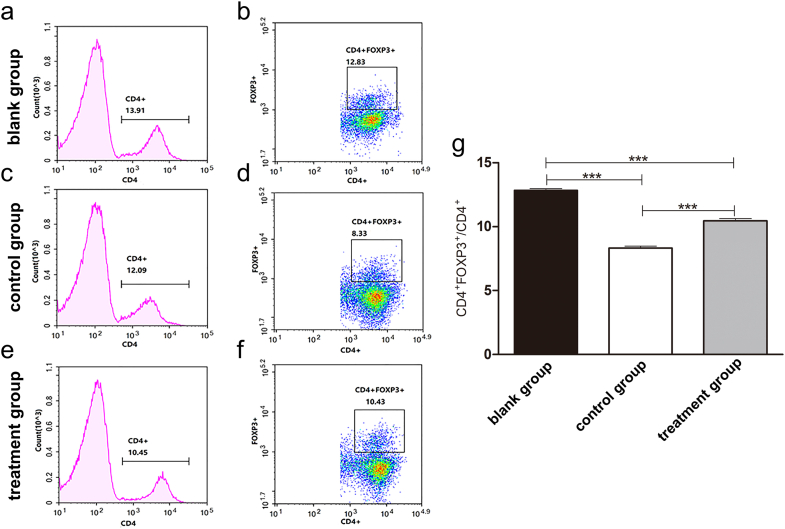
CD4^+^FOXP3^+^/CD4^+^ ratio in the spleen of control and treated mice. (a, b) Representative flow cytometry of CD4^+^FOXP3^+^/CD4^+^ ratio in blank group. (c, d) Representative flow cytometry of CD4^+^FOXP3^+^/CD4^+^ ratio in control group. (e, f) Representative flow cytometry of CD4^+^FOXP3^+^/CD4^+^ ratio in treatment group. (g) Comparisons of cell ratio between the three groups. Data are expressed as mean ± SD (five animals per group, n = 5). ***p < 0.001 by one-way analysis of variance.

To further investigate the molecular mechanism regarding the immunoregulatory role of FPS, the mRNA/protein expression of immune-related cytokines as well as ratio of immune cells were measured in control and treated mice. The results showed decreased mRNA expression levels of NF-κB (Fig. 5a), IL-6 (Fig. 5b), TNF-α (Fig. 5c), and iNOS (Fig. 5e) and increased mRNA expression level of COX-2 (Fig. 5d) in cyclophosphamide group. While in FPS treated group, these effects were reversed except for TNF-α. Moreover, cyclophosphamide decreased the protein expression of occludin and ZO-1 in cecal tissues and the CD4^+^FOXP3^+^/CD4^+^ ratio in spleen tissues (Fig. 5f–h; Fig. S6). On the contrary, these parameters showed increasing trends in FPS treated group (Fig. 6 e-g) compared with control group (Fig. 6c and d) and blank group (Fig. 6a and b). Collectively, the above results further revealed that FPS enhanced immunity by regulating immunoregulatory cytokines and immune cells.

### 3.5 Gut microbiota and immunity were closely correlated under FPS treatment in mice

Pearson correlation analysis between the mRNA expression of inflammatory genes, expression of tight junction proteins, and number of OTUs was performed. The results showed that the mRNA expression of IL-6 was significantly and positively correlated with the abundance of *Ruminococcaceae*_UCG-013 and *Christensenellaceae*_R-7_group and inversely correlated with the abundance of *Helicobacter*, *Anaerotruncus*, *Faecalibacterium*, *Lachnospira*, and *Erysipelotrichaceae*_UCG-003. The mRNA expression of COX-2 was significantly and positively associated with the abundance of *Helicobacter*, *Anaerotruncus*, *Mucispirillum*, *Faecalibacterium*, *Lachnospira*, and *Erysipelotrichaceae*_UCG-003 and inversely associated with the abundance of *Muribaculaceae*. The mRNA expression of NF-κB was significantly and positively correlated with the abundance of *Rhodospirillales*, *Ruminococcus*_1, and *Christensenellaceae*_R-7_group.

Occludin expression was significantly and positively associated with the abundance of *Christensenellaceae*_R-7_group and inversely correlated with the abundance of *Helicobacter*, *Anaerotruncus*, *Faecalibacterium*, *Lachnospira*, and *Erysipelotrichaceae*_UCG-003. The expression of ZO-1 was significantly and positively associated with the abundance of *Mollicutes*_RF39, *Muribaculaceae*, and *Ruminococcus*_1 and inversely related to the abundance of *Anaerotruncus* and *Mycoplasma* (Table 1).

**Table 1:** Pearson correlation analysis between cytokine mRNA expression, tight junction protein expression, and the number of operational taxonomic units.

Bacterial genus	Number of OTUs	NF-κB	IL-6	COX-2	Occludin	ZO-1
g_*Helicobacter*	003		−0.799**	0.702*	−0.872**	
g_*Anaerotruncus*	101		−0.730*	0.695*	−0.807**	−0.793*
g_*Mucispirillum*	005			0.781**		
g_*Mycoplasma*	030					−0.718*
g_*Faecalibacterium*	303		−0.757*	0.956**	−0.846**	
g_*Lachnospira*	322		−0.765**	0.962**	−0.853**	
g_*Erysipelotrichaceae*_UCG-003	368		−0.839**	0.679*	−0.889**	
g_*Rhodospirillales*	126	0.937**				
g_*Ruminococcaceae*_UCG-013	223		0.647*			
g_*Mollicutes*_RF39	265					0.840**
g_*Ruminococcus*_1	213	0.854**				0.679*
g_*Christensenellaceae*_R-7_group	244	0.772*	0.710*		0.633*	
g_*Muribaculaceae*	008			−0.643*		0.934**

*p < 0.05, **p < 0.01.

## 4 Discussion

In recent years, the polysaccharides in medicinal plants have attracted increasing attention due to their immunomodulatory effects. Specifically, studies have adopted immunosuppression models to investigate the immunomodulatory roles and mechanisms of polysaccharides. Several immunosuppressive agents, including dexamethasone, azathioprine, ethanol, and cyclophosphamide, have been widely used to establish immunosuppression models [[34], [35], [36]]. For instance, dexamethasone has been observed to decrease the number of all lymphocyte subpopulations in the blood of rabbits. In contrast, this drug had weaker effects on the number changes of lymphocytes in spleen, mesentery, and popliteal lymph nodes [36]. Ethanol caused the loss of thymocytes and splenocytes in mice, and the thymus recovered more slowly than the spleen after the interruption of ethanol feeding [35]. Cyclophosphamide, a cytotoxic immunosuppressive agent, significantly decreased the weight of the bursa of Fabricius in chickens [37] and decreased the number of white blood cells and lymphocytes in mice [38]. In our samples, cyclophosphamide decreased the spleen and thymus indices and the number of white blood cells and lymphocytes in the control group, indicating that immunosuppression was achieved in our model.

Cyclophosphamide induces cell cycle arrest [39] and apoptosis [40] and regulates the NF-κB pathway by controlling the dectin-1 and Toll-like receptor signaling pathways [41,42]. Medicinal plant polysaccharides enhanced immune responses in cyclophosphamide-treated mice [43]. For instance, polysaccharides from *Astragalus*, *Lycium barbarum*, and *Cordyceps militaris* stimulated the proliferation of spleen lymphocytes, reversed thymic and splenic damage, and reduced the features of immunosuppression in immunodeficient mice [[43], [44], [45]]. In our samples, FPS reduced the effects of cyclophosphamide by increasing the thymus and spleen indices, the number of white blood cells and lymphocytes, and the mRNA expression of NF-κB, IL-6, and iNOS, while downregulating COX-2 expression. These results are inconsistent with previous study which showed that wheat bran polysaccharides upregulated COX-2 and cytokine gene expression [46]. The reason may be that FPS downregulates the mRNA expression of the pro-inﬂammatory COX-2 (specifically by the regulation of ω-6 fatty acids), thereby improving immunological activities in mice [47]. Collectively, these data demonstrated that FPS improved the immunity of cyclophosphamide-treated mice.

It is now generally recognized that the intestinal microbiome can modulate host immunity [48]. Specifically, there is a complex interaction between gut microorganisms and host immune cells. Changes in bacterial composition favor the development of specific subtypes of lymphocytes, thereby affecting cytokine secretion [49]. This relationship is partly mediated by SCFAs including acetic, propionic, and butyric acids. SCFAs are functional metabolites produced by beneficial intestinal bacteria and have diverse regulatory functions in host physiology and immunity [50]. It is believed that SCFAs acidify the intestinal environment, protecting it against pathogenic bacteria [51]. In addition, SCFAs mediate the interaction between host cells and the intestinal epithelium, inhibit histone deacetylases, regulate immune processes (e.g., T cell differentiation), and improve intestinal epithelial barrier function [21,[52], [53], [54]].

Collectively, the mechanism by which FPS exert immune regulatory effects could be explained with the mechanism of action in two ways [[55], [56], [57]]. The first is that FPS metabolized to produce short chain fatty acids, thereby exerting immunoregulatory effects under the regulation of gut microbiota. The second is that FPS could regulate the intestinal microbiota structure of immunosuppressive mice and make its structure closer to normal mice, thereby exerting immunoregulatory effects by reducing harmful bacteria and increasing the proportion of beneficial bacteria. In our study, FPS decreased the relative abundance of *Helicobacter*, *Anaerotruncus*, *Faecalibacterium*, *Lachnospira*, *Erysipelotrichacea*e_UCG-003, *Mucispirillum*, and *Mycoplasma* (*p* < 0.05) and increased the relative abundance of *Rhodospirillales*, *Ruminococcaceae*_UCG-013, *Mollicutes*_RF39, *Ruminococcus*_1, *Christensenellaceae*_R-7_group, and *Muribaculaceae* (*p* < 0.05). *Helicobacter*, *Anaerotruncus*, *Erysipelotrichaceae*, and *Mucispirillum* are opportunistic pathogens and may induce inflammatory gene expression [[58], [59], [60], [61], [62]]. For instance, *Helicobacter* induces COX-2 expression by activating the epidermal growth factor receptor [58]. Chaihu-Shugan-San decoction improved intestinal dysbiosis by decreasing the abundance of *Enterobacteriaceae*, *Staphylococcaceae*, and *Veillonella* and increasing the expression of proinflammatory cytokines in a rat model of nonalcoholic fatty liver disease [63]. The increase in the abundance of *Ruminococcus*_1 and *Muribaculaceae* by noni fruit polysaccharide improved colonic barrier integrity by increasing the relative mRNA expression of ZO-1 and occludin in rats fed a high-fat diet [64]. *Mollicutes*_RF9 and *Ruminococcus*_1 reduced intestinal inflammation and increased the expression of tight junction proteins ZO-1 and occludin in high-fat diet-fed mice supplemented with milk fat globule membrane [65]. In the present study, FPS increased the concentrations of SCFAs in the intestine, suggesting that FPS increases the abundance of SCFA-producing bacteria. Similarly, resistant starch from lotus seeds increased the concentrations of SCFAs in mice by increasing the abundance of SCFA-producing bacteria, such as *Ruminococcaceae* [66]. In our model, changes in bacterial abundance correlated with the host immune profile. The abundance of *Rhodospirillales* was positively correlated with NF-κB expression, while the abundance of *Ruminococcaceae*_UCG-013 and *Christensenellaceae*_R-7_group was positively associated with IL-6 expression. The abundance of *Helicobacter* was positively correlated with COX-2 expression and inversely associated with occludin expression. In addition, the abundance of *Ruminococcus*_1, *Muribaculaceae*, and *Mollicutes*_RF9 was positively correlated with the protein expression of ZO-1. These results indicate that FPS increased the relative abundance of beneficial bacteria, improving immune function and intestinal barrier integrity.

## 5 Conclusion

In this study, we first revealed the effects of FPS and its mechanism in immunosuppressed mouse models. We found that FPS had the function of elevating the concentration of acetic acid, propionic acid, isobutyric acid, and n-butyric acid in the cecum. Meanwhile, FPS could decrease the relative abundance of *Helicobacter*, *Anaerotruncus*, *Faecalibacterium*, *Lachnospira*, *Erysipelotrichaceae*_UCG-003, *Mucispirillum*, and *Mycoplasma*, and increase the relative abundance of *Rhodospirillales*, *Ruminococcaceae*_UCG-013, *Mollicutes*_RF39, *Ruminococcus*_1, *Christensenellaceae*_R-7_group, and *Muribaculaceae* in the gut. Furthermore, FPS exhibited the function of increasing spleen and thymus indices and number of white blood cells and lymphocytes. FPS could reverse the decreased mRNA expression of NF-кB, IL-6, and iNOS, differentiation of CD4^+^FOXP3^+^ regulatory T cells as well as protein expression of occludin and ZO-1 induced by cyclophosphamide. In addition, gut microbiota and expression of immunoregulatory cytokines were closely correlated under FPS treatment in mice. Collectively, the above results demonstrated that FPS could regulate inflammatory cytokines and gut microbiota composition of immunosuppressive mice to improve immunity, thereby shedding light on revealing the molecular mechanism of polysaccharides of traditional Chinese medicines in the future.

## Author contribution statement

Keke Chen: conceived and designed the experiments; Wenfeng Huang: conceived and designed the experiments; Ran Tu: performed the experiments; analyzed and interpreted the data; wrote the paper. Cheng Zhou: performed the experiments; analyzed and interpreted the data; wrote the paper. Qiufang Zhao: contributed reagents, materials, analysis tools or data. Xiaofei Shi: contributed reagents, materials, analysis tools or data. Langjun Cui: contributed reagents, materials, analysis tools or data. Zhengping Feng: analyzed and interpreted the data; wrote the paper. Contributed reagents, materials, analysis tools or data; Qiufang Zhao, Xiaofei Shi, Langjun Cui. Wrote the paper: Ran Tu, Cheng Zhou, Zhengping Feng.

## Data availability statement

Data included in article/supp. material/referenced in article.

## Additional information

Supplementary content related to this article has been publish online at [URL].

## Funding

This research was funded by the Traditional Chinese Medicine Scientific Research Project of the Hubei Provincial Health Commission (Grant No. ZY2019Q035), Central University Innovation Team Project (Grant No. GK202001006, GK202006003), Shaanxi Province Key Research and Development Program (Grant No. 2019ZDLSF04-01-01, 2020SF-314), Special Project of Xi'an Science and Technology Plan Innovation Fund (Grant No. 2019KJWL04), and Shaanxi Aerospace Breeding Engineering Center Project (Grant No. 2021008).

## Declaration of competing interest

The authors declare that they have no known competing financial interests or personal relationships that could have appeared to influence the work reported in this paper.

## References

[1] Kennedy P.G.E. (2021). An overview of viral infections of the nervous system in the immunosuppressed. J. Neurol..

[2] Andersen K.M., Bates B.A., Rashidi E.S., Olex A.L., Mannon R.B., Patel R.C., Singh J., Sun J., Auwaerter P.G., Ng D.K., Segal J.B., Garibaldi B.T., Mehta H.B., Alexander G.C., Haendel M.A., Chute C.G. (2022). Long-term use of immunosuppressive medicines and in-hospital COVID-19 outcomes: a retrospective cohort study using data from the National COVID Cohort Collaborative. Lancet Rheumatol.

[3] Ma H.-D., Deng Y.-R., Tian Z., Lian Z.-X. (2013). Traditional Chinese medicine and immune regulation. Clin. Rev. Allergy Immunol..

[4] Fei X., Lina W., Jiayang C., Meng F., Guodong W., Yaping Y., Langjun C. (2021). Variations of microbial community in Aconitum carmichaeli Debx. rhizosphere soilin a short-term continuous cropping system. J. Microbiol..

[5] Tong P., Wu C., Wang X., Hu H., Jin H., Li C., Zhu Y., Shan L., Xiao L. (2013). Development and assessment of a complete-detoxication strategy for Fuzi (lateral root of Aconitum carmichaeli) and its application in rheumatoid arthritis therapy. J. Ethnopharmacol..

[6] Wei H., Wu H., Yu W., Yan X., Zhang X. (2015). Shenfu decoction as adjuvant therapy for improving quality of life and hepatic dysfunction in patients with symptomatic chronic heart failure. J. Ethnopharmacol..

[7] Yu B., Cao Y., Xiong Y.-K. (2015). Pharmacokinetics of aconitine-type alkaloids after oral administration of Fuzi (Aconiti Lateralis Radix Praeparata) in rats with chronic heart failure by microdialysis and ultra-high performance liquid chromatography–tandem mass spectrometry. J. Ethnopharmacol..

[8] Fu M., Zhang X., Chen B., Li M., Zhang G., Cui L. (2021). Characteristics of isolates of pseudomonas aeruginosa and serratia marcescens associated with post-harvest Fuzi (Aconitum carmichaelii) rot and their novel loop-mediated isothermal amplification detection methods. Front. Microbiol..

[9] Adeyemi E.A., Adebisi Y.A., Babatunde A.O. (2021). Psychosocial impacts of chronic kidney disease and dialysis therapy. SciMedicine J.

[10] Khorasgani M.A., Nejad P.M., Bashi M.M.M., Hedayati M. (2019). Evaluation of mir-377-3p expression in patients with multiple sclerosis. SciMedicine J.

[11] Wei Y., Tang H.Q., Li X.H., Zhu X.Y. (2013). Effects of Fuzi Lizhong Decoction on immune cell factors in rats with spleen yang deficiency syndrome. Chin. J. Exp. Tradit. Med. Formulae.

[12] Yan J.J. (2015).

[13] Wang H., Qi X.Z., Jia W., Yu J., Yang K., Zhang X., Wang L. (2023). The immunoregulatory effect of aconite treatment on h22 tumor-bearing mice via modulating adaptive immunity and natural killer-related immunity. Evid.-Based Compl. Alt..

[14] Tang F., Liang S.Y., Chen F.L., Tang Q.F., Tan X.M. (2015). Study on material basis of MahuangFuziXixin decoction for anti-inflammation and immune suppression based on combined method of serum pharmacochemistry and serum pharmacology. China J. Chin. Mater. Med..

[15] Zhao C., Li M., Luo Y., Wu W. (2006). Isolation and structural characterization of an immunostimulating polysaccharide from fuzi. Aconitum carmichaeli, Carbohyd. Res..

[16] Miao Z.H., Liu J.S., Wang Y.L., Dong L.F., Han C.Z., Han Y.Q., Zheng X.L. (2007). Experimental study of the effect of acid Monkshood polysaccharide on the immunologic function in immunosuppressive mice. Hebei J. Tradit. Chin. Med..

[17] Gao L.L., Zeng S.P., Pan L.T. (2012). Induction of differentiation of dendritic cells derived from hepatocellular carcinoma by Fuzi polysaccharides. Chin. J. Clin. Oncol..

[18] Barrea L., Muscogiuri G., Frias-Toral E., Laudisio D., Pugliese G., Castellucci B., Garcia-Velasquez E., Savastano S., Colao A. (2021). Nutrition and immune system: from the Mediterranean diet to dietary supplementary through the microbiota. Crit. Rev. Food Sci. Nutr..

[19] Fan Y., Pedersen O. (2021). Gut microbiota in human metabolic health and disease. Nat. Rev. Microbiol..

[20] Yoo J.Y., Groer M., Dutra S.V.O., Sarkar A., McSkimming D.I. (2020). Gut microbiota and immune system interactions. Microorganisms.

[21] Rooks M.G., Garrett W.S. (2016). Gut microbiota, metabolites and host immunity. Nat. Rev. Immunol..

[22] Zhang J., Yang G., Wen Y., Liu S., Li C., Yang R., Li W. (2017). Intestinal microbiota are involved in the immunomodulatory activities of longan polysaccharide. Mol. Nutr. Food Res..

[23] Tang C., Sun J., Zhou B., Jin C., Liu J., Kan J., Qian C., Zhang N. (2018). Effects of polysaccharides from purple sweet potatoes on immune response and gut microbiota composition in normal and cyclophosphamide treated mice. Food Funct..

[24] Chen D., Yang X., Zheng C., Yang J., Tang X., Chen J., Shuai O., Xie Y. (2017). Extracts from Hericium erinaceus relieve inflammatory bowel disease by regulating immunity and gut microbiota. Oncotarget.

[25] Yang H., Yin T., Zhang S. (2015). Isolation, Purification, and characterization of polysaccharides from wide Morchella esculenta (l.) Pers. Int. J. Food Prop..

[26] Huo W., Feng Z., Hu S., Cui L., Qiao T., Dai L., Qi P., Zhang L., Liu Y., Li J. (2020). Effects of polysaccharides from wild morels on immune response and gut microbiota composition in non-treated and cyclophosphamide-treated mice. Food Funct..

[27] Liu P.-Y., Wu W.-K., Chen C.-C., Panyod S., Sheen L.-Y., Wu M.-S. (2018). Evaluation of compatibility of 16S rRNA V3V4 and V4 amplicon libraries for clinical microbiome profiling. bioRxiv (2020) 2020.2008.

[28] Klindworth A., Pruesse E., Schweer T., Peplies J., Quast C., Horn M., Glöckner F.O. (2012). Evaluation of general 16S ribosomal RNA gene PCR primers for classical and next-generation sequencing-based diversity studies. Nucleic Acids Res..

[29] Quast C., Pruesse E., Yilmaz P., Gerken J., Schweer T., Yarza P., Peplies J., Glöckner F.O. (2012). The SILVA ribosomal RNA gene database project: improved data processing and web-based tools. Nucleic Acids Res..

[30] Augereau C., Lemaigre F.P., Jacquemin P. (2016). Extraction of high-quality RNA from pancreatic tissues for gene expression studies. Anal. Biochem..

[31] Livak K.J., Schmittgen T.D. (2001). Analysis of relative gene expression data using real-time quantitative PCR and the 2−ΔΔCT method. Methods.

[32] Puzio I., Muszyński S., Dobrowolski P., Kapica M., Pawłowska-Olszewska M., Donaldson J., Tomaszewska E. (2021). Alterations in small intestine and liver morphology, immunolocalization of leptin, ghrelin and nesfatin-1 as well as immunoexpression of tight junction proteins in intestinal mucosa after gastrectomy in rat model. J. Clin. Med..

[33] Xu H.-M., Huang H.-L., Xu J., He J., Zhao C., Peng Y., Zhao H.-L., Huang W.-Q., Cao C.-Y., Zhou Y.-J., Zhou Y.-L., Nie Y.-Q. (2021). Cross-talk between butyric acid and gut microbiota in ulcerative colitis following fecal microbiota transplantation. Front. Microbiol..

[34] Wiseman A.C. (2016). Immunosuppressive medications. Clin. J. Am. Soc. Nephrol..

[35] Jerrells T.R., Smith W., Eckardt M.J. (1990). Murine model of ethanol-induced immunosuppression. Alcohol Clin. Exp. Res..

[36] Jeklova E., Leva L., Jaglic Z., Faldyna M. (2008). Dexamethasone-induced immunosuppression: a rabbit model. Vet. Immunol. Immunopathol..

[37] El-Abasy M., Motobu M., Nakamura K., Koge K., Onodera T., Vainio O., Toivanen P., Hirota Y. (2004). Preventive and therapeutic effects of sugar cane extract on cyclophosphamide-induced immunosuppression in chickens. Int. Imunopharmacol..

[38] Huyan X.-H., Lin Y.-P., Gao T., Chen R.-Y., Fan Y.-M. (2011). Immunosuppressive effect of cyclophosphamide on white blood cells and lymphocyte subpopulations from peripheral blood of Balb/c mice. Int. Immunopharm..

[39] Xi M., He W., Li B., Zhou J., Xu Z., Wu H., Zhang Y., Song D., Hu L., Lu Y., Bu W., Kong Y., Chen G., Chang S., Shi J., Zhu W. (2020). Novel cyclophosphamide of natural products osalmide and pterostilbene induces cytotoxicity and cell cycle arrest in diffuse large B-cell lymphoma cells. Acta Biochim. Biophys. Sin..

[40] Davidoff A.N., Mendelow B.V. (1993). Cell-cycle disruptions and apoptosis induced by the cyclophosphamide derivative mafosfamide. Exp. Hematol..

[41] Yang J., Lu Q., Liu W., Wan Z., Wang X., Li R. (2010). Cyclophosphamide reduces dectin-1 expression in the lungs of naive and Aspergillus fumigatus-infected mice. Med. Mycol..

[42] Cao L.-P., Du J.-L., Jia R., Ding W.-D., Xu P., Yin G.-J., Zhang T. (2021). Effects of cyclophosphamide on antioxidative and immune functions of Nile tilapia (Oreochromis Niloticus) via the TLR-NF-κB signaling pathway. Aquat. Toxicol..

[43] Ding Y., Yan Y., Chen D., Ran L., Mi J., Lu L., Jing B., Li X., Zeng X., Cao Y. (2019). Modulating effects of polysaccharides from the fruits of Lycium barbarum on the immune response and gut microbiota in cyclophosphamide-treated mice. Food Funct..

[44] Wang M., Meng X.Y., Yang R.L., Qin T., Wang X.Y., Zhang K.Y., Fei C.Z., Li Y., Hu Y.l., Xue F.Q. (2012). Cordyceps militaris polysaccharides can enhance the immunity and antioxidation activity in immunosuppressed mice. Carbohyd. Polym..

[45] Li W., Hu X., Wang S., Jiao Z., Sun T., Liu T., Song K. (2020). Characterization and anti-tumor bioactivity of astragalus polysaccharides by immunomodulation. Int. J. Biol. Macromol..

[46] Shen T., Wang G., You L., Zhang L., Ren H., Hu W., Qiang Q., Wang X., Ji L., Gu Z., Zhao X. (2017). Polysaccharide from wheat bran induces cytokine expression via the toll-like receptor 4-mediated p38 MAPK signaling pathway and prevents cyclophosphamide-induced immunosuppression in mice. Food Nutr. Res..

[47] Chen C. (2010). COX-2's new role in inflammation. Nat. Chem. Biol..

[48] Zhang Z., Tang H., Chen P., Xie H., Tao Y. (2019). Demystifying the manipulation of host immunity, metabolism, and extraintestinal tumors by the gut microbiome. Signal Transduct. Targeted Ther..

[49] Takiishi T., Fenero C.I.M., Câmara N.O.S. (2017). Intestinal barrier and gut microbiota: shaping our immune responses throughout life. Tissue Barriers.

[50] Kumar M., Babaei P., Ji B.Y., Nielsen J. (2016). Human gut microbiota and healthy aging: recent developments and future prospective. Nutr. Healthy Aging.

[51] Kim H.J., White P.J. (2009). In vitro fermentation of oat flours from typical and high β-glucan oat lines. J. Agric. Food Chem..

[52] Cummings J.H., Pomare E.W., Branch W.J., Naylor C.P., Macfarlane G.T. (1987). Short chain fatty acids in human large intestine, portal, hepatic and venous blood. Gut.

[53] Thorburn A.N., McKenzie C.I., Shen S., Stanley D., Macia L., Mason L.J., Roberts L.K., Wong C.H.Y., Shim R., Robert R., Chevalier N., Tan J.K., Mariño E., Moore R.J., Wong L., McConville M.J., Tull D.L., Wood L.G., Murphy V.E., Mattes J., Gibson P.G., Mackay C.R. (2015). Evidence that asthma is a developmental origin disease influenced by maternal diet and bacterial metabolites. Nat. Commun..

[54] Valenzano M.C., DiGuilio K., Mercado J., Teter M., To J., Ferraro B., Mixson B., Manley I., Baker V., Moore B.A., Wertheimer J., Mullin J.M. (2015). Remodeling of tight junctions and enhancement of barrier integrity of the CACO-2 intestinal epithelial cell layer by micronutrients. PLoS One.

[55] Widowati W., Prahastuti S., Hidayat M., Hasiana S.T., Wahyudianingsih R., Afifah E., Kusuma H.S.W., Rizal R., Subangkit M. (2022). Protective effect of ethanolic extract of jati belanda (Guazuma ulmifolia L.) by inhibiting oxidative stress and inflammatory processes in cisplatin-induced nephrotoxicity in rats. Pak. Vet. J..

[56] Girsang E., Ginting C.N., Lister I.N.E., Gunawan Ky, Widowati W. (2021). Anti-inflammatory and antiaging properties of chlorogenic acid on UV-induced fibroblast cell. PeerJ.

[57] Widowati W., Jasaputra D.K., Wargasetia T.L., Eltania T.F., Azizah A.M., Subangkit M., Lister I.N.E., Ginting C.N., Girsang E., Faried A. (2020). Apoptotic potential of secretome from interleukin-induced natural killer cells toward breast cancer cell line by transwell assay. HAYATI J. Biosci..

[58] Sierra J.C., Hobbs S., Chaturvedi R., Yan F., Wilson K.T., Peek J. Richard M., Polk D.B. (2013). Induction of COX-2 expression by Helicobacter pylori is mediated by activation of epidermal growth factor receptor in gastric epithelial cells. Am. J. Physiol.-Gastr. L..

[59] Berry D., Kuzyk O., Rauch I., Heider S., Schwab C., Hainzl E., Decker T., Müller M., Strobl B., Schleper C., Urich T., Wagner M., Kenner L., Loy A. (2015). Intestinal microbiota signatures associated with inflammation history in mice experiencing recurring colitis. Front. Microbiol..

[60] Kaakoush N.O. (2015). Insights into the role of erysipelotrichaceae in the human host. Front. Cell. Infect. Microbiol..

[61] Lee S.-M., Han H.W., Yim S.Y. (2015). Beneficial effects of soy milk and fiber on high cholesterol diet-induced alteration of gut microbiota and inflammatory gene expression in rats. Food Funct..

[62] Selvanantham T., Lin Q., Guo C.X., Surendra A., Fieve S., Escalante N.K., Guttman D.S., Streutker C.J., Robertson S.J., Philpott D.J., Mallevaey T. (2016). NKT cell–deficient mice harbor an altered microbiota that fuels intestinal inflammation during chemically induced colitis. J. Immunol..

[63] Liang Y., Zhang Y., Deng Y., Liang S., He Y., Chen Y., Liu C., Lin C., Han L., Tu G., Yang Q. (2018). Chaihu-Shugan-San decoction modulates intestinal microbe dysbiosis and alleviates chronic metabolic inflammation in NAFLD rats via the NLRP3 inflammasome pathway. Evid.-Based Compl. Alt. 2018.

[64] Yang X., Mo W., Zheng C., Li W., Tang J., Wu X. (2020). Alleviating effects of noni fruit polysaccharide on hepatic oxidative stress and inflammation in rats under a high-fat diet and its possible mechanisms. Food Funct..

[65] Li T., Gao J., Du M., Mao X. (2018). Milk fat globule membrane supplementation modulates the gut microbiota and attenuates metabolic endotoxemia in high-fat diet-fed mice. J. Funct.Foods.

[66] Zeng H., Huang C., Lin S., Zheng M., Chen C., Zheng B., Zhang Y. (2017). Lotus seed resistant starch regulates gut microbiota and increases short-chain fatty acids production and mineral absorption in mice. J. Agric. Food Chem..

