# Supplementary material for: Fuzi polysaccharides improve immunity in immunosuppressed mouse models by regulating gut microbiota composition
Source: Heliyon. 2023 Jul 13;9(7):e18244. doi: 10.1016/j.heliyon.2023.e18244 (PMC10372400; doi:10.1016/j.heliyon.2023.e18244)
Supplement: Multimedia component 1 [file mmc1.docx]

**Fuzi polysaccharides improve immunity in immunosuppressed mouse models by regulating gut microbiota composition**

**Supplementary Data**

**Table S1.** Primers used in quantitative real-time polymerase chain reaction.

| Gene | Primer sequences |
| --- | --- |
| *IL-6* | F GACAACCACGGCCTTCCCT |
|  | R GGTACTCCAGAAGACCAGAG |
| *TNF-α* | F AGCACACAGAAAGCATGAT |
|  | R CTGATGAGAGGGAGGCCATT |
| *i-NOS* | F TGGTGAAGGGACTGAGCTGTT |
|  | R GCACAAGGGGTTTTCTTCACG |
| *NF-κB* | F TCTCAGCTGCGACCCCGGA |
|  | R TGGGCTCAATGATCTCCTCT |
| *COX-2* | F ACCCTGCCTACGAAGGAAC |
|  | R ACCACGGTTTTGACATGGGT |
| *GAPDH* | F CGTCCCGTAGACAAAATGG |
|  | R TTGATGGCAACAATCTCCAC |

F: forward; R: reverse.


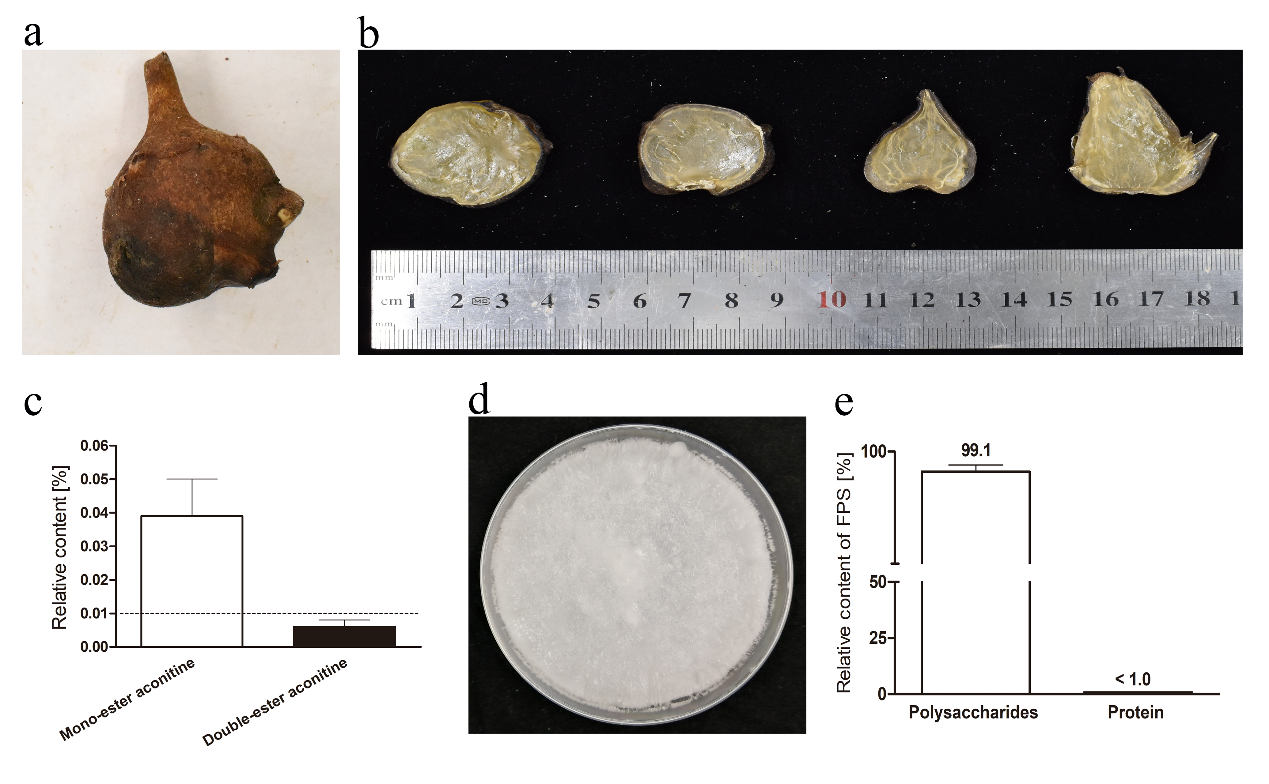


**Figure S1.** Preparation of Fuzi polysaccharides (FPS). (a) Fuzi roots. (b) Hei-Shun-Pian roots. Fuzi roots were processed into Hei Shun-Pian using traditional methods. (c) The relative concentrations of monoester aconitines (benzoylmesaconine, benzoylaconitine, and benzoylhypaconitine) and double ester aconitines (mesaconitine, aconitine, and hypaconitine) in Hei-Shun-Pian complied with Chinese pharmacopoeia requirements (2015 edition). Data were obtained from three biological replicates. (d) Freeze-dried FPS powder. (e) Relative concentration of polysaccharides and proteins in the FPS fraction. Data were obtained from three biological replicates.


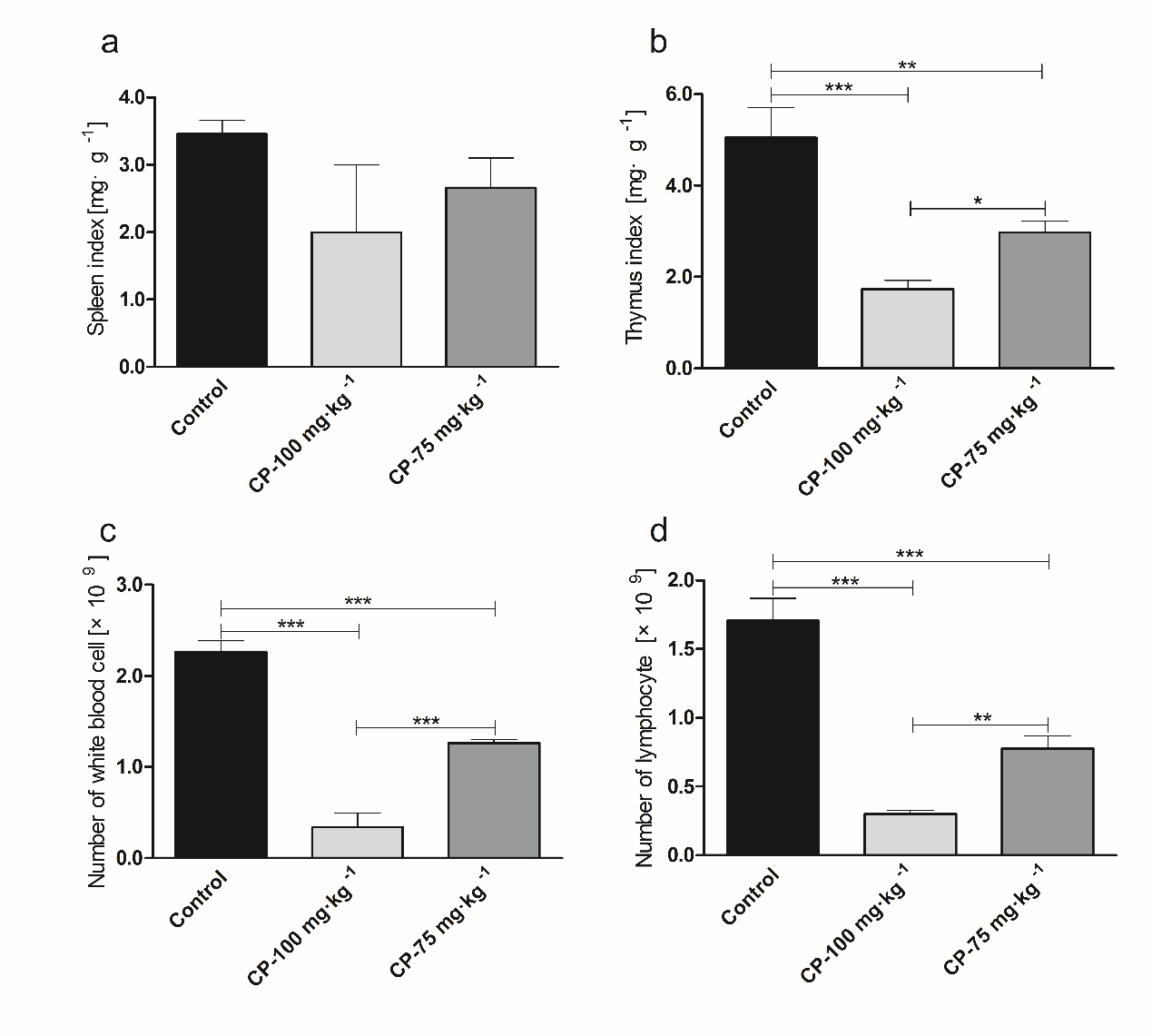


**Figure S2.** Effects of two concentrations of cyclophosphamide (CP) on immune organ indices. (a) spleen index. (b) thymus index. (c) number of white blood cells. (d) number of lymphocytes. Control mice were treated with sterile saline (0.2 mL). Mice were intraperitoneally injected with CP (75 or 100 mg·kg^-1^) for 3 days. The group treated with the lower dose was used to establish the mouse model. Data are expressed as mean ± SD (five animals per group, n=5). * *p* < 0.05, ** *p* < 0.01, *** *p* < 0.001 by one-way analysis of variance.


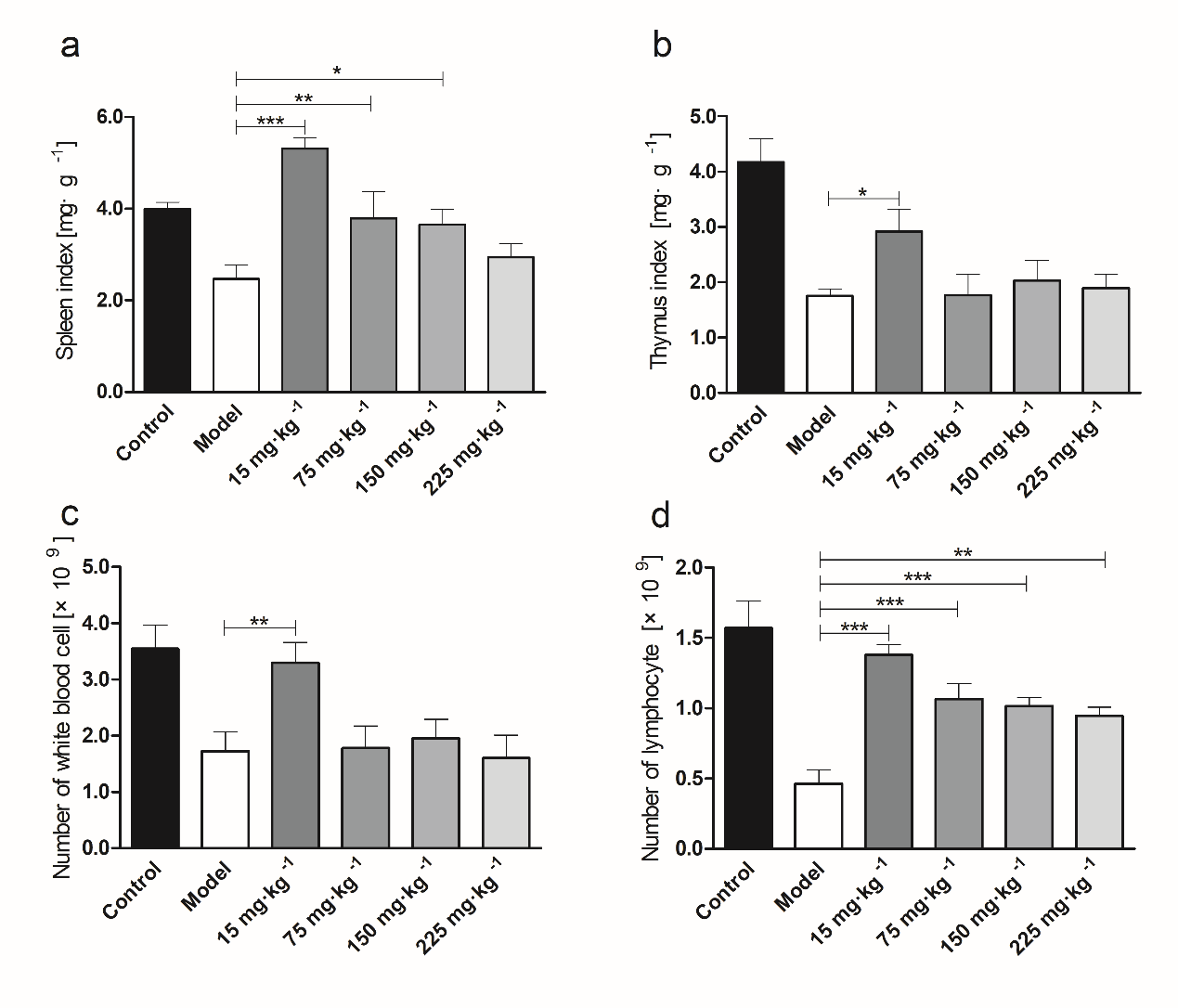


**Figure S3.** Spleen index (a), thymus index (b), number of white blood cells (c), and number of lymphocytes (d) in control and treated mice. Animals were divided into three groups of five animals: blank group, treated with sterile saline; control group, intraperitoneally injected with cyclophosphamide (CP, 75 mg·kg^-1^) for 3 days and gavaged with sterile saline for 7 days; treatment group, intraperitoneally injected with CP (75 mg·kg^-1^) for 3 days and gavaged with Fuzi polysaccharides (15, 75, 150, or 225 mg·kg^-1^) for 7 days. The lowest CP dose showed the strongest effect and was used in subsequent experiments. Data are expressed as mean ± SD (five animals per group, n=5). * *p* < 0.05, ** *p* < 0.01, *** *p* < 0.001 by one-way analysis of variance.


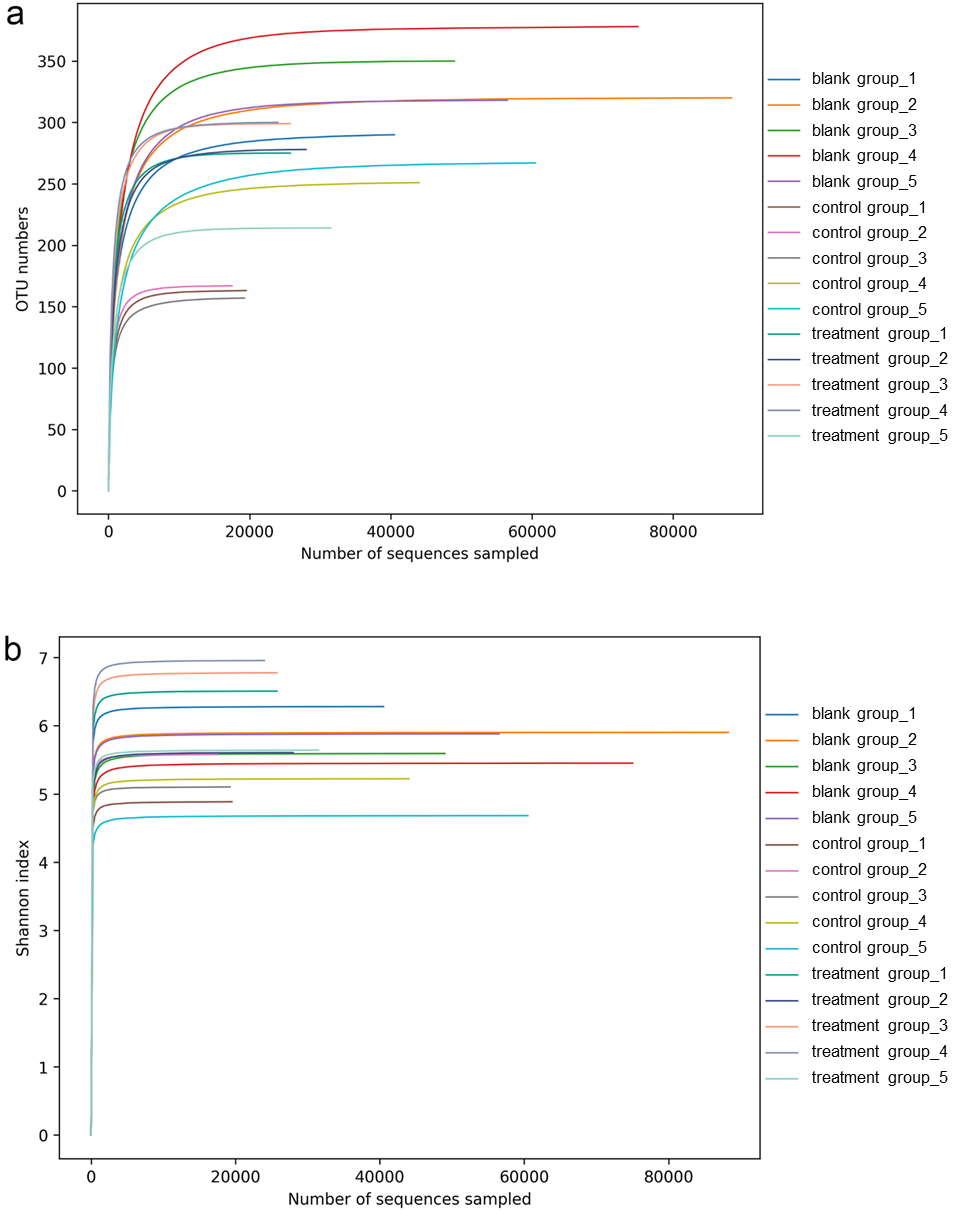


**Figure S4.** Rarefaction curves (a) and Shannon curves (b) of the mouse intestinal microbiota. Mice were assigned to three groups and treated as described in Figure S3 (five animals per group, n=5).


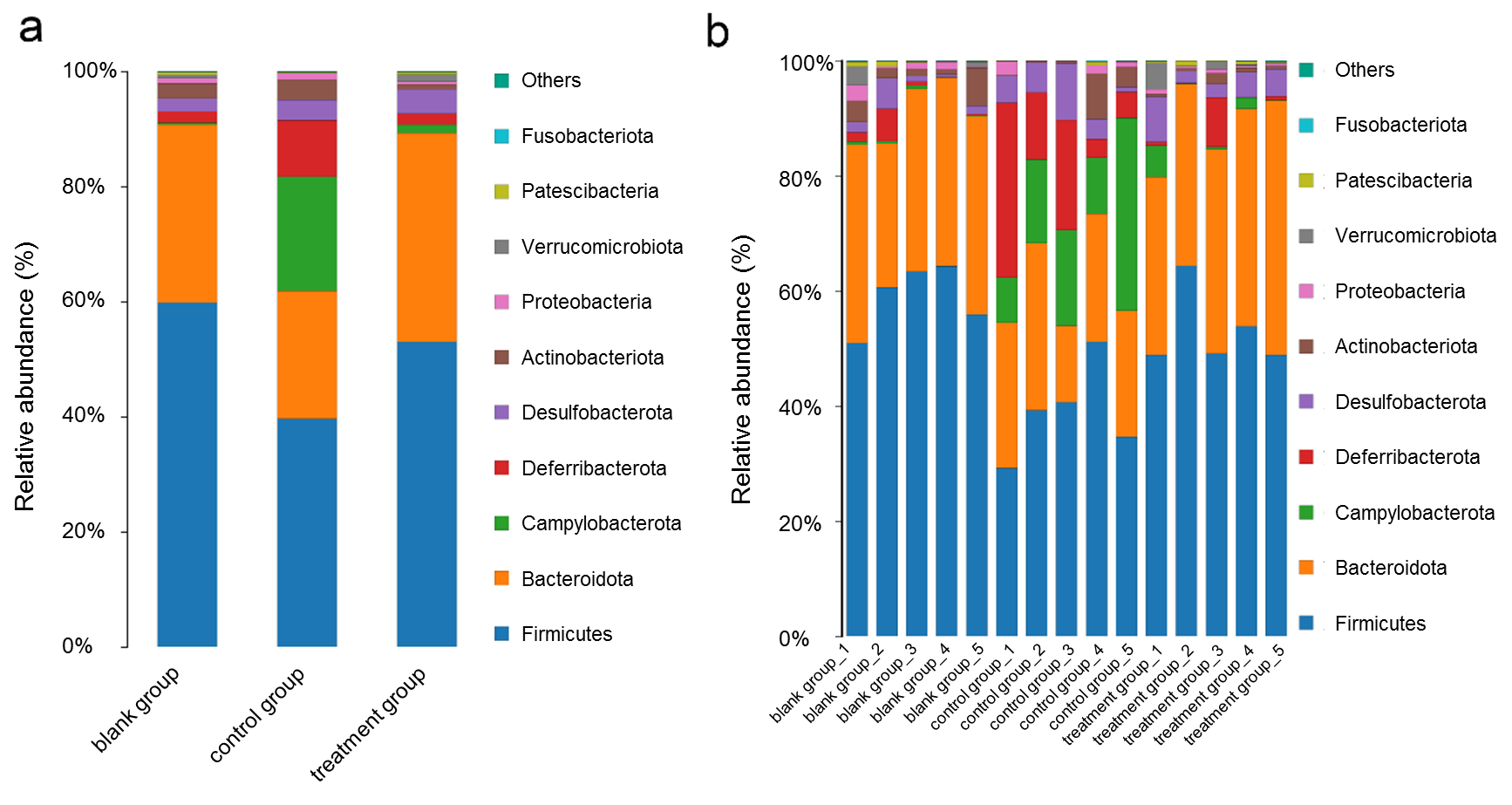


**Figure S5.** Abundance of intestinal bacteria at the phylum level. Mice were assigned to three groups and treated as described in Figure S2 (five animals per group, n=5). (a) Gut microbial composition of each group at the phylum level. (b) Gut microbial composition of each sample in each group at the phylum level. The ten most abundant phyla are shown.


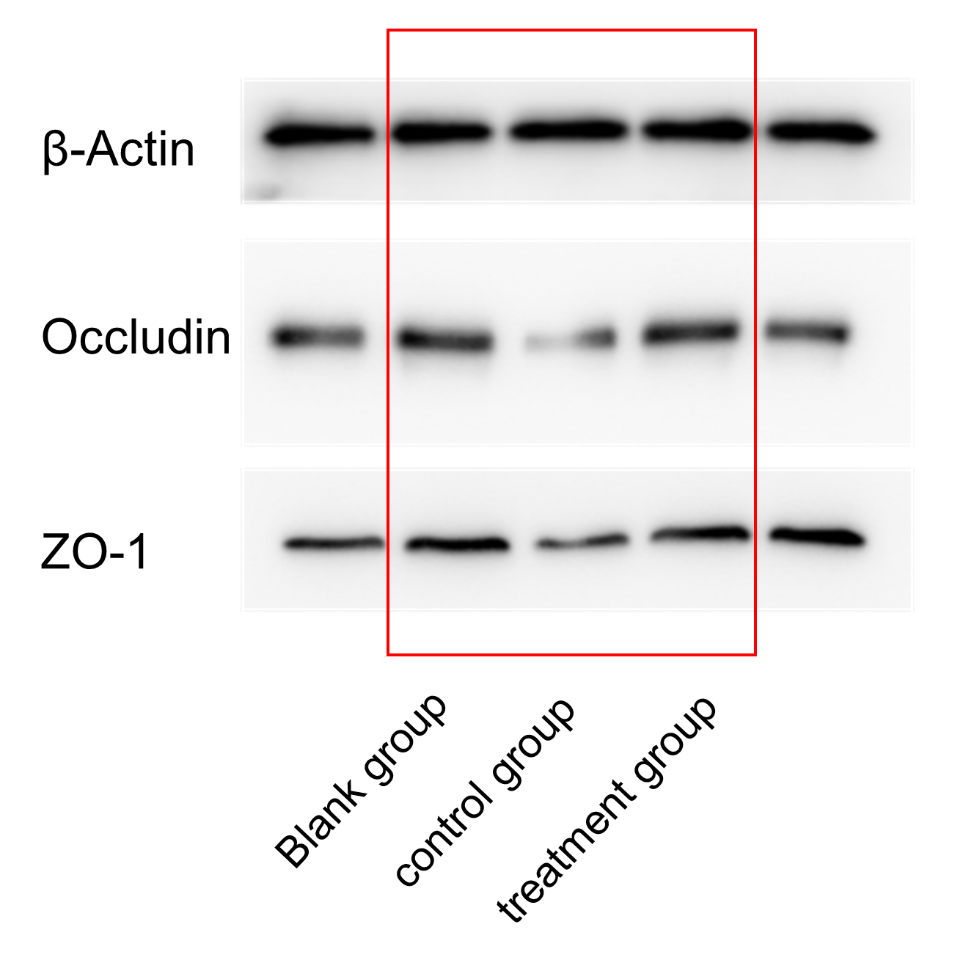


**Figure S6.** Uncropped versions of Figure 5f. Representative western blotting images of tight junction proteins (ZO-1 and occludin) were originated from the above uncropped gel. The other bands were protein expression under treatments of Fuzi slices (left-most) and Fuzi extracts (right-most).
